# Risk factors predisposing to cardia gastric adenocarcinoma: Insights and new perspectives

**DOI:** 10.1002/cam4.2497

**Published:** 2019-08-25

**Authors:** Esmat Abdi, Saeid Latifi‐Navid, Saber Zahri, Abbas Yazdanbod, Farhad Pourfarzi

**Affiliations:** ^1^ Department of Biology Faculty of Sciences University of Mohaghegh Ardabili Ardabil Iran; ^2^ Digestive Diseases Research Center Ardabil University of Medical Sciences Ardabil Iran

**Keywords:** cardia gastric adenocarcinoma, *Helicobacter pylori*, risk biomarkers

## Abstract

Recent decades have seen an alarming increase in the incidence of cardia gastric adenocarcinoma (CGA) while noncardia gastric adenocarcinoma (NCGA) has decreased. In 2012, 260 000 CGA cases (age‐standardised rate (ASR); 3.3/100 000) and 691 000 NCGA cases (ASR; 8.8/100 000) were reported worldwide. Compared with women, men had greater rates for both the subsites, especially for CGA. Recently, four molecular subtypes of GC have been proposed by the Cancer Genome Atlas (TCGA) and the Asian Cancer Research Group (ACRG); however, these classifications do not take into account predisposing germline variants and their possible interaction with somatic alterations in carcinogenesis. The etiology of adenocarcinoma of the cardia and the gastroesophageal junction (GEJ) is not known. It is thought that CGA is distinct from adenocarcinomas located in the esophagus or distal stomach, both epidemiologically and biologically. Moreover, CGA is often identified in the advanced stage having a poor prognosis. Therefore, understanding the risk and the role of predisposing factors in etiology of CGA can inform clinical practice and counseling for risk reduction. In this paper, we showed that GC family history, lifestyle, demographics, gastroesophageal reflux disease, *Helicobacter pylori* infection, and multiple genetic and epigenetic risk factors as well as several predisposing conditions may underlie susceptibility to CGA. However, several genome‐wide association studies (GWASs) should be conducted to identify novel high‐penetrance genes and pathways as well as causal germline variants predisposing to CGA. They must include different ethnic groups, especially from high‐incidence countries for CGA, because some risk loci are ancestry‐specific. In parallel, statistical methods can be developed to identify cancer predisposition genes (CPGs) from tumor sequencing data. It is also necessary to find novel long noncoding RNAs related to the risk of CGA. Taken altogether, new cancer risk prediction models, including all genetic and nongenetic factors influencing risk, should be developed to facilitate risk assessment, disease prevention, and early diagnosis and intervention of CGA in the future.

## Graphical abstract

The etiology of adenocarcinoma of the cardia and the gastroesophageal junction (GEJ) is not known and is doubted. It is thought that cardia gastric adenocarcinoma (CGA) is distinct from adenocarcinomas located in the esophagus or distal stomach, both epidemiologically and biologically. Moreover, CGA is often identified in the advanced stage having a poor prognosis. In this paper, we would like to ascertain the possible role of GC family history, lifestyle, demographics, gastroesophageal reflux disease, *Helicobacter pylori* infection, and multiple genetic and epigenetic risk factors as well as several predisposing conditions in susceptibility to CGA.

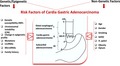

## 1 INTRODUCTION

Gastric cancer (GC) is the fifth common cancer (6.8%) in the world and the third leading cause of death related to cancer (8.8%) worldwide.^1^ In fact, the complicated interaction between *Helicobacter pylori* (*H pylori*) infection and genetic, epigenetic, and environmental factors results in GC.^2^ Gastric adenocarcinoma is the prominent type of GC, which is classified into two major histological subtypes of intestinal and diffuse adenocarcinoma according to Lauren's classification, reflecting its pathogenesis.^3^ There are two GC subtypes, cardia (occurring in the 1‐cm (cm) proximal and 2‐cm distal area of the esophago‐gastric junction) gastric adenocarcinoma (CGA) and noncardia (distal: involving the distal and middle parts of the stomach) gastric adenocarcinoma (NCGA).^4^ In 2012, 260 000 CGA cases (age‐standardised rate (ASR) 3.3 per 100 000) and 691 000 NCGA cases (ASR 8.8) were reported all over the world. The greatest regional rates of both GC subsites were in Eastern/Southeastern Asia (in men, ASRs: 8.7 and 21.7 for CGA and NCGA, respectively). NCGA was observed more commonly than CGA with a mean ratio of 2:1 in most countries, but in some populations, the rates of NCGA incidence were less than the global mean.^5^ Ardabil Province in Northwest of Iran has the highest CGA rates in the world. In Ardabil, over one‐third of the GC occurs in the cardia region of the stomach having only 5%‐10% of the whole stomach, and the ASRs for CGA are 26.4 and 8.6 for males and females, respectively.^6^

The etiology of adenocarcinoma of the cardia and the gastroesophageal junction (GEJ) is not known and is doubted. It is thought that CGA is distinct from adenocarcinomas located in the esophagus or distal stomach, both epidemiologically and biologically.^7^ Moreover, CGA is often identified in the advanced stage having a poor prognosis. In this paper, we would like to ascertain the possible role of GC family history, lifestyle, demographics, gastroesophageal reflux disease, *H pylori* infection, and multiple genetic and epigenetic risk factors as well as several predisposing conditions in susceptibility to CGA. Therefore, understanding risk and the role of these factors in etiology of CGA can inform clinical practice and counseling for risk reduction.

## 2 FAMILY HISTORY

Most GCs are sporadic; however, nearly 10% represents familial aggregation with an unclear molecular basis. Hereditary cancers constitute less than 3% of all stomach cancers and are recessed into the three autosomal dominant syndromes: hereditary diffuse GC (HDGC), familial intestinal GC, and gastric adenocarcinoma and proximal polyposis of the stomach.^8^ HDGC is the most commonly known familial GC and is characterized by CDH1 deletion. However, it is rare, not taking into account a large proportion of family clustering.^9^ The incidence rate of HDGC in the cardia and noncardia subsites of the stomach is also not clear.

Family history of GC raises the risk of its development, with risks ranging from 1.3 to 3.0 for the first‐degree relatives of GC cases. GC development under 50 years of age is probably followed by family history.^10^ People with a positive paternal family history were at higher risk of GC compared to positive maternal family history.^11^ Coexistence of two risk factors including a positive family history and infection with a CagA‐positive *H pylori* isolate could increase more than 16‐fold risk of NCGA and eightfold total risk of CGA.^12^ Thus, identifying inherited parameters among subjects with GC family histories is an important step for due diagnosis and management of the disease.

## 3 DEMOGRAPHIC AND BEHAVIORAL FACTORS

The GC incidence increases with age. The median age for GC diagnosis is 70.^13^ Compared with women, men had greater rates for both the subsites, especially for CGA (male‐to‐female ratio 3:1).^5^ This marked difference is likely to be due to endogenous factors, such as reproductive hormones, different prevalence of central obesity between two sexes, or different premenopausal iron status. However, it cannot be explained by different smoking histories.^14^ Estrogen—the female sex hormone—is a suppressor of the inflammatory response and cytokine production in certain tissues, thus likely having similar effects in the upper gastrointestinal (GI) tract. In addition, lower body iron stored during their reproductive years in females might change the degree of DNA damage caused by chronic inflammation. Male predominance of upper GI adenocarcinomas is also related to the intestinal subtype rather than tumor subsite because of delayed development of this subtype in females before 50‐60 years.^15^

A meta‐analysis study revealed that smoking was associated with CGA and the relative risk (RR) was 1.87. RR rose from 1.3 for the lowest intake to 1.7 for about 30 cigarettes per day.^16^ Risks of CGA were higher than those of NCGA in former, moderate, and high‐intensity cigarette smokers.^17^ It also relates opium use to a higher risk of GC^18^ with an augmented CGA risk (OR = 2.8).^19^ The obesity prevalence, indicated by body mass index (BMI ≥30 kg/m^2^), has increased over the past two decades. Fat is metabolically active and generates many compounds that move in the body. These products (eg, insulin‐like growth factor and leptin) are related to malignancies, probably via inducing pro‐growth changes in the cycle of a cell, declined cell death, and pro‐neoplastic cellular variations.^20^ Meta‐analysis showed that risen BMI correlated with the CGA risk (CGA, summary relative risk, SRRs = 1.21 and 1.82 for overweight and obesity, respectively, but not with NCGA (NCGA; SRRs = 0.93 and 1.00 for overweight and obesity, respectively.^21^ A meta‐analysis revealed a 21% decline in GC risk, in those having higher physical activity compared to the least active ones. This risk decline was reported for both NCGA (37% risk reduction) and CGA (20% risk reduction).^22^

## 4 GASTROESOPHAGEAL REFLUX DISEASE

Gastroesophageal reflux disease (GERD), troublesome and recurrent heartburn and regurgitation, is known as a primary risk factor for upper gastrointestinal cancers. Significant associations have been found between CGA and GERD, with two‐ to fourfolds of increased risk in many studies; however, not all studies confirm it.^23^, ^24^ The increase in the occurrence of CGA in the Western world was elaborated by increasing GERD incidence and obesity.^25^ CGA was related with gastric atrophy (OR = 3.92) and GERD symptoms (OR = 10.08), hence results show two different etiologies of CGA, one resulting from intense atrophic gastritis (intestinal or diffuse subtype) as NCGA and another from GERD (intestinal subtype).^23^, ^26^ Endoscopic screening of men with chronic GERD symptoms (≥5 years) who have at least two additional risk factors (eg age >50 years, central obesity, past or current history of smoking, White race, or family history of Barrett esophagus) is suggested by current guidelines.^27^ However, there are junctional cancers in patients who never had typical reflux diseases, largely explained by two entities of partial hiatus hernia and intrasphincteric reflux.^28^ Hiatal hernia (HH) is a significant independent risk factor for CGA and esophageal adenocarcinoma. HH in combination with reflux symptoms was strongly associated with the risk of esophageal adenocarcinomas (OR = 8.11). This association was more modest for CGA (OR = 2.93).^29^ It has also been shown that in the asymptomatic, moderately overweight population with no reflux, there are cardiac mucosal lengthening and proximal extension of gastric acid within the lower esophageal sphincter, thus likely causing the observed change in the cardiac mucosa. These changes may be related to the etiology of CGA and GEJ, often seen in people without a history of reflux disease.^30^, ^31^

## 5 *Helicobacter pylori* INFECTION

The main risk factor of intestinal metaplasia, chronic atrophic gastritis, and gastric adenocarcinoma is *H pylori* that colonizes the human stomach.^32^ Studies on Asian countries have revealed a higher positive association between *H pylori* infection and CGA, while some other studies of Western countries have reported no association or even inverse association.^33^, ^34^ The meta‐analysis provided evidence for a positive association between CGA and *H pylori* infection. For CGA, summary RR was 1.08 (95% CI 0.83‐1.40), greater in high‐risk (RR = 1.98; 95% CI 1.38‐2.83) than in low‐risk situations (RR = 0.78; 95% CI 0.63‐0.97).^35^ Individual antigen testing has revealed that CagA positivity is associated with an increased risk of CGA and NCGA, which is in line with other studies conducted in Asian populations.^36^ The *vacA* c1 genotype of *H pylori* has strongly increased the risk of CGA (OR = 14.11). *H pylori vacA* c1 genotype is also thought to be the primary bacterial biomarker for the prediction of CGA risk in Iranian males aged >55.^37^ In contrast, the *vacA* c2 genotype, particularly in combination with *cag*PAI genotypes (ie *cagH*, *cagL*, *cagG*, and *orf17*), showed strong inverse associations with the risk of CGA and non‐CGA, indicating a coordinated relationship between the *vacA* c2 and *cag*PAI genotypes.^38^

## 6 GENETIC RISK FACTORS

### 6.1 New molecular subtypes of GC

Recently, four molecular subtypes of GC have been determined by the Cancer Genome Atlas (TCGA) project, which include Epstein‐Barr virus (EBV), microsatellite instability (MSI), genomically stable (GS), and chromosomal instability (CIN).^39^ CIN subtype, which mostly occurs in the esophago‐gastric junction (EGJ)/cardia, represents at least 50% of GCs.^40^ It is related to intestinal‐type histology, showing elevated frequency in the EGJ/cardia, according to TCGA characterization (65%).^41^ Furthermore, the Asian Cancer Research Group (ACRG) has proposed other molecular classification, including mesenchymal subgroup (MSS/EMT), microsatellite instability subgroup (MSI), Microsatellite Stable *TP53*‐positive (MSS/TP53^+^, corresponding to EBV^+^ subtype by TCGA), and Microsatellite Stable *TP53*‐negative tumors (MSS/TP53^−^, corresponding to CIN subtype by TCGA). Microsatellite‐unstable tumors, which occur in the antrum, are hypermutated intestinal‐subtype tumors having the best prognosis and the lowest frequency of recurrence (22%) of the four subtypes. The mesenchymal‐like type, including diffuse‐subtype tumors, which have the tendency to occur at an earlier age, shows the worst prognosis and the highest recurrence frequency (63%) of the four subtypes.^42^

These classifications open new horizons for identification of relevant genomic subsets for precision oncology using highly complex methodologies, including genomic screening and molecular, epigenetic, and functional characterization. However, the two classifications have some limitations. They lack a prospective validation on a large scale, including patients from other geographic regions of the world. The differences between them are greater than similarities, which include differences in molecular mechanisms, relation to prognosis, and the distribution of Lauren's diffuse subtype among the four subgroups. Neither of them considers active and nonmalignant stromal cells. Stromal gene expression profiles may influence assignment to a specific subtype. On the other hand, novel stromal‐based signatures have been related to the dominant cancer phenotypes. Thus, the classification of GC can be improved from a tumor stroma perspective.^43^, ^44^, ^45^

Although these subtypes may be related to the prognosis of GC patients and determine the patient's benefits from adjuvant chemotherapy after large‐scale validation trials, they do not take into account predisposing inherited germline variants for cancer. Recent data have shown that somatic cancer genes also show recessive rare, damaging germline variants (RDGVs) that predispose to cancer via a two‐hit mechanism.^46^ This indicates a possible interaction of the germline variants with somatic driver alterations in carcinogenesis. For example, germline variants in RBFOX1, a gene encoding an RNA‐binding protein involved in splicing, increase the incidence of SF3B1 somatic mutation by eightfold. Similarly, 19p13.3 variants are associated with a fourfold increase in somatic mutation rate of the PTEN tumor suppressor gene.^47^ However, the impact of large‐scale tumor sequencing has been limited in identifying cancer predisposition genes (CPGs).

### 6.2 Single‐nucleotide polymorphisms in CGA

Single‐nucleotide polymorphisms (SNPs) are natural genetic changes occurring with different frequencies in various populations. Some SNPs may change the gene expression profile and influence function of the gene, leading to risen susceptibility risk to the range of some disorders, like cancer. There are many instances of polymorphic genes, which raise the susceptibility to GC.

#### 6.2.1 PRKAA1

One SNP, rs10074991 in PRKAA1 at 5p13.1, reached genome‐wide significance for CGA. PRKAA1 protein is a catalytic subunit of AMP‐activated protein kinase (AMPK), crucial for the regulation of cellular energy metabolism. To respond to the decline of intracellular ATP levels, AMPK stimulates energy‐production pathways and prevents processes of energy consuming leading to the inhibition of biosynthesis of protein, carbohydrate, and lipid, and prevention of cell growth and proliferation.^48^

#### 6.2.2 MUC1 and PLCE1

The glycoprotein Mucin 1 is aberrantly glycosylated and overexpressed in epithelial cancers, and plays an important role in disease progression.^49^ Phospholipase C epsilon‐1 (PLCE1) is a phospholipase C isoenzyme encoded by PLCE1 gene, it interacts with the proto‐oncogene Ras among other proteins. PLCE1‐related signaling network affects many critical carcinogenetic processes like metabolism, proliferation, survival, and tumor growth. In a genome‐wide association study (GWAS) conducted among Chinese people, positive correlations among SNPs in MUC1 and CGA and NCGA were similar. Two independent GWAS datasets in Chinese showed associations between multiple variants at 10q23, on gene PLCE1, and CGA risk.^50^, ^51^

#### 6.2.3 NF‐κBs

NF‐κBs are stimulated in many cancers, the equivalent of “nonclassical oncogene.” The combined effect analysis revealed that when carrying the *NFKBIA* gene polymorphism site of rs696 (AA) and *NFKB1* gene polymorphism site of rs3755867 (GG), the CGA incidence risk was more than the time the adverse genotype (OR = 5.22) was not carried.^52^

#### 6.2.4 IL1B‐31C, IL1B‐511T, and IL1RN2

Non‐Asian populations also showed augmented risks among IL1B‐31C, IL1B‐511T, and IL1RN2 carriers for CGA, but this was not significant in Asian populations.^53^

#### 6.2.5 P27 (kip1)

The p27kip1 expression is an early event in gastric tumorigenesis, and is regarded as a candidate molecular biomarker for early GC.^54^ P27 (kip1) polymorphisms may be associated with the CGA susceptibilities in North China.

#### 6.2.6 MTHFR

The enzyme methylenetetrahydrofolate reductase (MTHFR) has an important role in the regulation of methionine and homocysteine concentrations in folate metabolism.^55^ Individuals with the MTHFR 677TT variant genotype possessed a twofold increased CGA risk (OR = 2.04).^56^

#### 6.2.7 ADPRT

A study showed ORs of 2.17 and 1.61 for CGA in the ADPRT (Adenosine diphosphate ribosyl transferase) Ala/Ala or XRCC1 (X‐ray repair cross‐complementing 1) Gln/Gln genotype carriers, respectively, compared to noncarriers. Gene‐gene interaction of XRCC1 and ADPRT polymorphisms raised the OR of CGA in a hasty manner (OR for the combined XRCC1 Gln/Gln and ADPRT Ala/Ala genotypes was 6.43).^57^

#### 6.2.8 COX‐2

COX‐2, a major enzyme converting arachidonate to prostaglandins, is not present in normal cells unless quickly stimulated by different carcinogens. The level of COX‐2 was considerably increased in gastrointestinal cancer.^58^ Multivariate logistic regression analysis showed that the −1195AA, −765GC, and 587Arg/Arg genotypes of COX‐2 were related with increased CGA risk (OR = 1.50, OR = 2.06, and OR = 1.67, respectively). These results showed that the functional polymorphisms of COX‐2, when interacting with smoking, have an influential impact on developing CGA.^59^

#### 6.2.9 MDM2

Some epidemiological studies have found an association between murine double minute 2 (MDM2) SNP309 and the risk of different cancer types. TP53 induces intracellular expression of MDM2, whereas the latter induces the downregulation of TP53, the auto‐regulatory feedback loop between TP53 and MDM2. The relationship between MDM2 SNP309 and GC risk was meaningful, especially in CGA for the *H pylori*‐positive population group.^60^ Genotype analyses demonstrated that increased risk for development of CGA was correlated with the MDM2 309G and the P53 72Pro allele compared to the P53 72Arg allele and the MDM2 309T in an allele dose‐dependent manner.^61^

#### 6.2.10 RANK

Overexpression of receptor activator of nuclear factor κ B (RANK) directly induces epithelial‐to‐mesenchymal transition and stem‐like phenotypes in tumor cells and normal mammary epithelial cells. The RANK/ RANKL/OPG system, mechanistically, affects tumor cell invasion and migration.^62^ RANK rs1805034 T>C correlates with susceptibility to CGA, which is more obvious in elderly patients, male patients, smokers, and patients with no alcohol consumption.^63^

#### 6.2.11 PD‐1

Programmed cell death‐1 (PD‐1) is a major preventer of antitumor responses; it is a cogent candidate for genetic risk of subjects to many malignancies. Two ligands of PD‐1, programmed death‐1 ligand 1 (PD‐L1) and PD‐L2, inhibit activation and proliferation of T cells, leading to tumor escape from immune surveillance.^64^ A considerable increased risk of CGA related with the PD‐1 rs2227982 C>T polymorphism was observed among ever drinking subjects (TT vs CC: OR = 2.53, TT+CT vs CC: OR = 2.04).^65^ According to *TCGA*, *PD‐L1* gene was frequently amplified in EBV‐positive GC, probably indicating the higher immunogenicity of this GC subclass. Amplification of a chromosomal region 9p24.1 (locus of PD‐L1 and PD‐L2) has been seen at 15% of EBV‐positive GC.^66^

#### 6.2.12 MYT1

MYT/NZF family transcription factors include two major members, myelin transcription factor 1 (MYT1, or neural zinc finger 2 (NZF2)) and its homologue MYT1‐like (MYT1L or NZF1); each of them has six copies of a ZnF including a C_2_HC consensus sequence. MYT1 is also related with carcinoma.^67^ MYT1L rs17039396 variants could be a suitable prognostic indicator for GC, especially among the CGA.^68^

#### 6.2.13 XPG

XPG gene (or ERCC5) affects the excision of an *24‐32 bp DNA segment having the bulky adduct in nucleotide excision repair (NER). The T/T genotype of XPG and rs751402 C/T SNP T allele was correlated with an increased CGA risk in younger subjects (≤61 years; OR = 1.33). The T/T genotype carriers must receive periodic upper gastrointestinal endoscopy to facilitate the early diagnosis and cure of CGA.^69^

#### 6.2.14 MMP‐2

Matrix metalloproteinase‐2 (MMP‐2) is mainly responsible for regulating inflammatory response.^70^ People with the CC genotype of MMP‐2 had >threefold augmented risk (OR = 3.36) for development of CGA in comparison to those with the variant CT or TT genotype.^71^ MMP‐2 C−1306T polymorphism is a risk factor for CGA and the multifactor interactions among polymorphisms in FASL, MMP‐2, and FAS affect the CGA development.^72^ The detailed information regarding the genetic factors of CGA are indicated in Table 1.

**Table 1:** Role of genetic factors in CGA

	Case/control	*P*‐value	OR (95% CI)	Ref.
PRKAA1 (rs10074991)	3042/7548	7.36 × 10^−12^	0.83 (0.79‐0.88)	[ 48]
MUC‐1				[ 50]
rs4072037 (A>G)	1213/3302	9.5 × 10^−5^	0.75 (0.62‐0.87)	
rs4460629 (C>T)		1.3 × 10^−4^	0.74 (0.64‐0.86)	
PLCE1				[ 50, 51]
rs2274223 (A>G)	2766/ 11013	1.7 × 10^−39^	1.55 (1.45‐1.66)	
rs2274223 (A>G)		4.2 × 10^−15^	1.57 (1.40‐1.76)	
rs3765524 (C>T)	1213/3302	7.4 × 10^−15^	1.56 (1.40‐1.75)	
rs3781264 (T>C)		1.1 × 10^−13^	1.60 (1.41‐1.81)	
rs11187842 (C>T)		7.1 × 10^−12^	1.63 (1.42‐1.87)	
rs753724 (G>T)		8.0 × 10^−12^	1.63 (1.42‐1.87)	
NFKBIA (rs696 AA)	NA	<.05	5.22 (1.10, 24.92)	[ 52]
NFKB1 (rs3755867 GG)				
P27(kip1) V/V	256/437	<.05	2.56 (1.06‐4.78)	[ 54]
MTHFR‐ 677TT	217/468	<.05	2.04 (1.28‐3.26)	[ 56]
ADPRT (Ala/Ala)	500/1000	.017	2.17 (1.55‐3.04)	[ 57]
XRCC1 (Gln/Gln)		<.0001	1.61 (1.06 ‐2.44)	
COX‐2				[ 59]
1195AA	357/985	.038	1.50 (1.05‐2.13)	
765GC		.009	2.06 (1.29‐3.29)	
587Arg/Arg		.033	1.67 (1.04‐2.66)	
MDM2 ‐309				[ 60]
GG vs TT	999/2322	<.05	2.00 (1.61‐2.50)	
GT vs TT			1.50 (1.20‐1.88)	
RANK (rs1805034 T>C)				[ 63]
TC vs TT	323/592	.026	NR	
CC vs TT		.0003	NR	
TC/CC vs TT		.0019	NR	
CC vs TT/TC		.002	NR	
PD‐1 (rs2227982 C>T)				[ 65]
TT vs CC	330/608	.028	2.53 (1.11‐5.79)	
TT+CT vs CC		.047	2.04 (1.01‐4.13)	
MYT1L (rs17039396 GG)	174/90	.001	NR	[ 68]
XPG (rs751402)				[ 69]
C/T	212/216	<.05	1.33 (1.00‐1.76)	
T/T		.05	1.77 (1.12‐3.30)	
MMP2 −1306CC	356/789	<.05	3.36 (2.34‐4.97)	[ 71]
MMP‐2 −1306CC				
FASL‐ 844TT or TC	344/324	<.05	4.58 (2.07‐10.14)	
FAS‐ 1377AA				[ 72]

Abbreviations: NA, not available; NR, not reported; SNP, single‐nucleotide polymorphism.

## 7 EPIGENETIC RISK FACTORS

Promoter CpG island hypermethylation is popular in human cancers and correlates with transcriptional silencing of the associated gene.^73^ RASSF1A is placed on 3p21.3 and regulates apoptosis, cell cycle, microtubule stability, and other physiological activities. Epigenetic silencing of RASSF1A gene expression through promoter hypermethylation affects CGA. The RASSF1A gene's promoter methylation increased the CGA risk significantly (OR = 7.50).^74^ The CpG island hypermethylation at the promoter region of HLTF has also been found in the colon and stomach cancers, manifesting that aberrant methylation of HLTF affects carcinogenesis. HLTF methylation may be present in gastric cardia dysplasia phases and may affect the CGA development in subjects with a family history of UGIC.^75^ The impact of TSP1 on cancer progression is still controversial and shows stimulatory and inhibitory effects. Epigenetic silencing of TSP1 gene via promoter hypermethylation can affect CGA.^76^ CAV1 may regulate multiple intracellular signaling pathways. CAV1 expression loss with aberrant promoter methylation was detected in some human cancers. The CpG island shore methylation of CAV1 possibly affects the CGA progression and is a prognostic methylation biomarker for CGA cases.^77^

The loss of p16 (INK4A) protein expression can be detected in 45% of cardiac, esophageal, and gastric adenocarcinoma and correlates with p16 (INK4A) gene hypermethylation. Methylation of CpG in the EBV‐positive class is even greater than that in the MSI class. Moreover, viral cancers have a unique pattern of downregulation‐related methylation of CDKN2A (p16). Hypermethylation of p16 (INK4A) is a common research outcome in CGA.^78^ The proximal promoter aberrant hypermethylation and MEG3 enhancer region were seen in tissues of CGA. Also, the enhancer region and proximal promoter hypermethylation and dysregulation of MEG3 and miR‐770 were correlated with a survival of poorer CGA patients.^79^ Aberrant hypermethylation‐mediated downregulation of C5orf66‐AS1 may play critical roles in CGA tumorigenesis and C5orf66‐AS1 can be a prognostic marker in the prediction of CGA patients' survival.^80^ Epigenetic silencing of Wnt‐antagonist gene expression via promoter hypermethylation can influence CGA.^81^

Being land of E‐cadherin gene, high methylation status of 5' CPG may be a mechanism in developing CGA.^82^ A recent study indicated that there were a lot of males with CGA characterized by higher GATA5 DNA methylation values.^83^ FBXO32 (atrogin‐1) is an Fbox protein family member and has one of the four subunits of the ubiquitin protein ligase complex, contributing to muscle atrophy.^84^ Aberrant hypermethylation of FBXO32 is a mechanism resulting in loss or downexpression of the gene in CGA. FBXO32 is assumed as a functional tumor suppressor, and FBXO32 gene reactivation may have a therapeutic potential, indicating its role as a prognostic marker for CGA cases.^85^ It is demonstrated that the loss of RKIP expression and hypermethylation can be regarded as a marker to anticipate clinical result of CGA. It is suggested that RKIP is a new candidate gene among metastasis suppressors.^86^ The detailed information regarding the epigenetic factors of CGA are indicated in Table 2.

**Table 2:** Role of epigenetic factors in CGA

	Case/control	*P*‐value	OR (95% CI)	Ref.
RASSF1A	92/30	<.001	7.50 (2.78‐20.23)	[ 74]
HLTF	96/96	<.05	NR	[ 75]
TSP1	96/96	<.001	NR	[ 76]
CAV1	172/172	<.001	NR	[ 77]
p16^INK4A^	50/50	.002	NR	[ 78]
MEG3	134/134	<.001	NR	[ 79]
C5orf66‐AS1	125/125	<.001	NR	[ 80]
Wnt‐antagonist genes				
sFRP1	94/94	.000	NR	[ 81]
sFRP 2		.001	NR	
sFRP 4		.000	NR	
sFRP 5		.000	NR	
Wif‐1		.000	NR	
Dkk3		.000	NR	
E‐cadherin	92/92	<.001	NR	[ 82]
GATA5	105/105	<.05	NR	[ 83]
FBXO32	139/139	<.001	NR	[ 85]
RKIP	145/145	.000	NR	[ 86]
miR‐25/miR‐93/miR‐106b				
rs1534309	107/1284	5.38 × 10^−3^	0.56 (0.37‐0.86)	[ 87]
rs2070215		.0421	1.37 (5 1.02‐1.85)	

Abbreviations: NA, not available; NR, not reported; SNP, single‐nucleotide polymorphism.

## 8 LONG NONCODING RNAs

Long noncoding RNAs (lncRNAs) are transcribed RNAs longer than 200 nt which lack an open reading frame of considerable length. lncRNAs are expressed at lower levels compared to mRNAs. lncRNAs’ ectopic expression influences the GC development.^88^ There are not many articles on the variations of lncRNAs and the risk of CGA development. Notable downregulation of LOC100130476 was observed in primary CGA tissues, and SGC‐7901 and BGC‐823 cell lines. LOC100130476 can function as a tumor inhibitor gene in carcinogenesis of CGA. Aberrant methylation at the CpG sites next to the transcription start site within exon 1 might be important for gene silencing. LOC100130476 ectopic expression is considered a new biomarker for the early diagnosis of GC.^89^ C5orf66‐AS1 was considerably downregulated in cell lines and CGA tissues, and the level of expression was correlated with lymph node metastasis, pathological differentiation, TNM stage, and distant metastasis or recurrence.^90^ Table 3 shows the results obtained from microarray analysis of lncRNAs in CGA.

**Table 3:** Role of ncRNAs in promoting CGA

	Expression changes	Case/control	*P*‐value	Fold change (log2)	Ref.
LncRNAs					
C5orf66‐AS1	Downregulated	125/125	<.01	NA	[ 80]
LOC100130476	Downregulated	121/121	.013	1.907 (1.148‐3.166)^a^	[ 89]
ASHG19A3A028863	Upregulated	12/12	<.05	169.6730934	[ 90]
ASHG19A3A040903	Upregulated			41.90954829	
ASHG19A3A041865	Upregulated			39.16918169	
ASHG19A3A018727	Upregulated			28.88943866	
ASHG19A3A052295	Upregulated			24.55914831	
GUST‐20‐P1426265844	Upregulated			22.40102966	
ASHG19A3A041043	Upregulated			20.64951965	
ASHG19A3A033911	Upregulated			15.82403426	
ASHG19A3A026346	Upregulated			15.43079683	
ASHG19A3A007184	Downregulated			59.38580626	
ASHG19A3A018598	Downregulated			15.16286445	
ASHG19A3A038967	Downregulated			9.499758688	
ASHG19A3H0000023	Downregulated			9.473660683	
ASHG19A3A018662	Downregulated			9.338922844	
ASHG19A3A007413	Downregulated			8.588461452	
ASHG19A3A011053	Downregulated			7.817390602	
ASHG19A3A035937	Downregulated			7.2417301	
ASHG19A3A055173	Downregulated			5.954896947	
ASHG19A3A0001119	Downregulated			4.960711075	
Micro RNAs					
miR‐770	Downregulated	134/134	<.01	NR	[ 79]
miR‐141	Downregulated	41/41	<.05	NR	[ 91]
miR‐203a	Downregulated	127/127	.033	1.77 (1.046‐3.011)^a^	[ 92]
miR‐107 (rs2296616 TC/CC)	Upregulated	NA	NR	1.49 (1.01‐2.20)^b^	[ 93]
miR‐3656	Downregulated	21/21	1.89E−16	−3.29535	[ 94]
miR‐378c	Downregulated		8.96E−14	−1.80765	
miR‐628‐3p	Downregulated		2.23E−13	−2.03238	
miR‐US33‐3p	Downregulated		2.67E−13	−2.25544	
miR‐148a‐3p	Downregulated		2.67E−13	−1.63085	
miR‐H10	Downregulated		4.43E−13	−2.84551	
miR‐638	Downregulated		8.99E−13	−1.55968	
miR‐483‐5p	Downregulated		2.20E−12	−1.35334	
miR‐675‐5p	Downregulated		5.11E−12	−1.70156	
miR‐1184	Downregulated		2.67E−11	−1.00147	
miR‐299‐5p	Downregulated		3.05E−11	−1.66357	
miR‐4285	Downregulated		4.74E−11	−1.06365	
miR‐3665	Downregulated		9.57E−11	−1.95478	
miR‐H25	Downregulated		1.04E−10	−1.61128	
miR‐H17	Downregulated		1.41E−10	−1.53334	
miR‐3195	Downregulated		1.41E−10	−1.28305	
miR‐518e‐5p	Downregulated		1.41E−10	−0.97021	
miR‐3196	Downregulated		7.06E−10	−2.64801	
miR‐30d‐5p	Downregulated		7.06E−10	−0.74407	
miR‐3124‐5p	Downregulated		2.21E−09	−2.60563	
miR‐196a‐5p	Upregulated		3.36E−14	4.111534	
miR‐135b‐5p	Upregulated		2.67E−13	2.555514	
miR‐2355‐3p	Upregulated		2.68E−13	1.517697	
miR‐4307	Upregulated		1.05E−09	2.371521	
miR‐1244	Upregulated		3.68E−09	2.409671	
miR‐892a	Upregulated		1.05E−08	1.8554	
miR‐20a‐5p	Upregulated		1.15E−08	1.501549	
miRPlusA1087	Upregulated		6.38E−08	2.115592	
miR‐93‐5p	Upregulated		1.06E−07	1.5392	
miR‐455‐3p	Upregulated		1.80E−07	1.568063	
miR‐105‐5p	Upregulated		1.96E−07	1.755387	
miR‐764	Upregulated		2.58E−07	1.650002	
miR‐130b‐5p	Upregulated		4.98E−07	1.660447	
miR‐506‐3p	Upregulated		2.66E−06	1.605885	
miR‐454‐3p	Upregulated		3.92E−06	1.515466	
miR‐142‐3p	Upregulated		4.35E−06	1.524762	
miR‐3591‐3p	Upregulated		1.19E−05	1.452323	
miR‐196b‐5p	Upregulated		1.67E−05	1.682773	
miR‐3664‐5p	Upregulated		4.36E−05	1.737875	
miR‐636	Upregulated		9.98E−05	1.557929	

Abbreviations: NA, not available; NR, not reported.

a OR (95% CI).

b Hazard ratio (HR).

## 9 MICRORNAS

MicroRNAs (miRNAs) are single‐stranded small (20‐22 nt) ncRNAs which regulate gene expression and contribute to a broad spectrum of biological processes like cell proliferation, differentiation, apoptosis, endothelial cell migration, and angiogenesis.^95^ Some studies reported that miR‐141 was decreased and correlated with lymph node metastases in CGA and advanced TNM stage. Additionally, miR‐141 may stop cell proliferation and trigger apoptosis in adenocarcinoma gastric cell line. Also, miR‐141 may directly stop MACC1 through binding to its 3'‐UTR. It can affect the signaling pathways of MEK/ERK and p38 MAPK. It is a potential therapeutic goal for treating CGA cases.^91^ MEG3 and miR‐770 were notably downregulated in CGA patients and correlated with lymph node metastasis and TNM stage. The aberrant hypermethylation of the proximal promoter and MEG3 enhancer region was observed in CGA.^79^ Two tagSNPs of cluster 7.1 (miR‐25/miR‐93/miR‐106b) were found to be related with the GC cardia localization, rs2070215 (OR = 1.37) and rs1534309 (OR = 0.56).^87^ Significant downregulation and proximal promoter methylation of miR‐203b and miR‐203a in CGA were observed in CGA tissue. CGA cases in stage III and IV with decreased expression or hypermethylation of miR‐203a showed weak survival. MiR‐203b and miR‐203a may act as tumor suppressive miRNAs,miR‐203a reactivation may be regarded as a prognostic marker for CGA subjects.^92^ MiR‐107 is dysregulated in CGA pathogenesis, and the SNP rs2296616 may affect the process.^93^ It was found that four miRNAs (ie, miR‐3196, miR‐1244, miR‐135b‐5p, and miR‐628‐3p) were associated with differentiation of CGA. The miR‐196a‐5p was correlated with age of CGA onset. Survival analysis revealed that the miR‐135b‐5p expression level was correlated with survival of CGA.^94^ Table 3 presents the results obtained from microarray analysis of miRNAs in CGA.

## 10 CONCLUSION

CGA is a multi‐factorial ailment and most cases are sporadic, although familial cases have been reported. There is much difference between CGA and NCGA in terms of tumor features, distinct etiological factors, and biological behaviors. Lifestyle, *H pylori* infection, GERD, and multiple genetic, epigenetic, and environmental risk factors have been related to an increased risk of CGA. However, several GWASs, followed by a large‐scale GWAS meta‐analysis, should be conducted to identify novel high‐penetrance genes and pathways as well as causal germline variants predisposing to CGA. They must include different ethnic groups, especially from high‐incidence countries for CGA, because some risk loci are ancestry‐specific.^96^, ^97^ In parallel, statistical methods can also be developed to identify CPGs from tumor sequencing data. Then, it should be largely explored how the genetic germline variants and somatic alterations interact to develop CGA in populations with different ethnic backgrounds. A little experiment has also been done on the impact of lncRNAs on the carcinogenesis of the CGA. Therefore, next‐generation high‐throughput RNA‐sequencing techniques can enable us to find novel ncRNA biomarkers related to the risk of CGA. Taken altogether, new cancer risk prediction models, including all genetic and nongenetic factors influencing risk should be developed to facilitate risk assessment, disease prevention, and early diagnosis and intervention of CGA in the future.

## CONFLICT OF INTEREST

None declared.

## AUTHOR CONTRIBUTIONS

EA, SLN, and SZ provided direction in the preparation of the manuscript. EA and SLN performed primary literature research. EA and SLN wrote the first draft of manuscript. SZ, AY, and FP discussed and revised the manuscript. EA, AY, and FP managed the references. SLN approved the version to be published.

## References

[1] Ferlay J , Soerjomataram I , Dikshit R , et al. Cancer incidence and mortality worldwide: sources, methods and major patterns in GLOBOCAN 2012. Int J Cancer. 2015;136:E359‐E386. 25220842 10.1002/ijc.29210

[2] Chmiela M , Karwowska Z , Gonciarz W , Allushi B , Staczek P . Host pathogen interactions in Helicobacter pylori related gastric cancer. World J Gastroenterol. 2017;23:1521‐1540. 28321154 10.3748/wjg.v23.i9.1521 PMC5340805

[3] Lauren P . The two histological main types of gastric carcinoma: diffuse and so‐called intestinal‐type carcinoma. An Attempt at a histo‐clinical classification. Acta Pathol Et Microbiolo Scand. 1965;64:31‐49. 10.1111/apm.1965.64.1.31 14320675

[4] Odze RD , Riddell RH , Bosman FT , et al. Premalignant lesions of the digestive system In: Bosman FT, Carneiro F, Hruban RH, Theise ND, eds. WHO Classification of Tumours of the Digestive System. 4th edn Lyon, France: IARC; 2010.

[5] Colquhoun A , Arnold M , Ferlay J , Goodman KJ , Forman D , Soerjomataram I . Global patterns of cardia and non‐cardia gastric cancer incidence in 2012. Gut. 2015;64:1881‐1888. 25748648 10.1136/gutjnl-2014-308915

[6] Malekzadeh R , Sotoudeh M , Derakhshan M , et al. Prevalence of gastric precancerous lesions in Ardabil, a high incidence province for gastric adenocarcinoma in the northwest of Iran. J Clin Pathol. 2004;57:37‐42. 14693833 10.1136/jcp.57.1.37 PMC1770167

[7] Wang L‐D , Zheng S , Zheng Z‐Y , Casson AG . Primary adenocarcinomas of lower esophagus, esophagogastric junction and gastric cardia: in special reference to China. World J Gastroenterol. 2003;9:1156. 12800215 10.3748/wjg.v9.i6.1156 PMC4611775

[8] Oliveira C , Pinheiro H , Figueiredo J , Seruca R , Carneiro F . Familial gastric cancer: genetic susceptibility, pathology, and implications for management. Lancet Oncol. 2015;16:e60‐e70. 25638682 10.1016/S1470-2045(14)71016-2

[9] McLean MH , El‐Omar EM . Genetics of gastric cancer. Nat Rev Gastroenterol Hepatol. 2014;11:664‐674. 25134511 10.1038/nrgastro.2014.143

[10] Hemminki K , Sundquist J , Ji J . Familial risk for gastric carcinoma: an updated study from Sweden. Br J Cancer. 2007;96:1272. 17406355 10.1038/sj.bjc.6603722 PMC2360151

[11] Bakir T , Can G , Siviloglu C , Erkul S . Gastric cancer and other organ cancer history in the parents of patients with gastric cancer. Eur J Cancer Prev. 2003;12:183‐189. 12771555 10.1097/00008469-200306000-00003

[12] Chen M‐J , Wu D‐C , Ko Y‐C , Chiou Y‐Y . Personal history and family history as a predictor of gastric cardiac adenocarcinoma risk: a case‐control study in Taiwan. Am J Gastroenterol. 2004;99:1250. 15233662 10.1111/j.1572-0241.2004.30872.x

[13] Howlader N , Noone A , Krapcho M , et al. SEER cancer statistics review, 1975‐2008. Bethesda, MD: National Cancer Institute; 2011 Based on November 2010 SEER data submission, posted to the SEER website, http://seer.cancer.gov/csr/1975_2008. Accessed June, 8

[14] Freedman ND , Derakhshan MH , Abnet CC , Schatzkin A , Hollenbeck AR , McColl KE . Male predominance of upper gastrointestinal adenocarcinoma cannot be explained by differences in tobacco smoking in men versus women. Eur J Cancer. 2010;46:2473‐2478. 20605442 10.1016/j.ejca.2010.05.005 PMC3514413

[15] Derakhshan MH , Liptrot S , Paul J , Brown IL , Morrison D , McColl KE . Oesophageal and gastric intestinal‐type adenocarcinomas show the same male predominance due to a 17 year delayed development in females. Gut. 2009;58:16‐23. 18838486 10.1136/gut.2008.161331

[16] Ladeiras‐Lopes R , Pereira AK , Nogueira A , et al. Smoking and gastric cancer: systematic review and meta‐analysis of cohort studies. Cancer Causes Control. 2008;19:689‐701. 18293090 10.1007/s10552-008-9132-y

[17] Praud D , Rota M , Pelucchi C , et al. Cigarette smoking and gastric cancer in the Stomach Cancer Pooling (StoP) Project. Eur J Cancer Prev. 2018;27:124‐133. 27560662 10.1097/CEJ.0000000000000290

[18] Sadjadi A , Derakhshan MH , Yazdanbod A , et al. Neglected role of hookah and opium in gastric carcinogenesis: a cohort study on risk factors and attributable fractions. Int J Cancer. 2014;134:181‐188. 23797606 10.1002/ijc.28344 PMC5821120

[19] Shakeri R , Malekzadeh R , Etemadi A , et al. Opium: an emerging risk factor for gastric adenocarcinoma. Int J Cancer. 2013;133:455‐461. 23319416 10.1002/ijc.28018 PMC3644384

[20] Alemán JO , Eusebi LH , Ricciardiello L , Patidar K , Sanyal AJ , Holt PR . Mechanisms of obesity‐induced gastrointestinal neoplasia. Gastroenterology. 2014;146:357‐373. 24315827 10.1053/j.gastro.2013.11.051 PMC3978703

[21] Chen Y , Liu L , Wang X , et al. Body mass index and risk of gastric cancer: a meta‐analysis of a population with more than ten million from 24 prospective studies. Cancer Epidemiol Biomark Prev. 2013;22:1395‐1408. 10.1158/1055-9965.EPI-13-0042 23697611

[22] Singh S , Varayil JE , Devanna S , Murad MH , Iyer PG . Physical activity is associated with reduced risk of gastric cancer: a systematic review and meta‐analysis. Cancer Prev Res. 2014;7:12‐22. 10.1158/1940-6207.CAPR-13-0282 24048713

[23] Derakhshan MH , Malekzadeh R , Watabe H , et al. Combination of gastric atrophy, reflux symptoms and histological subtype indicates two distinct aetiologies of gastric cardia cancer. Gut. 2008;57:298‐305. 17965056 10.1136/gut.2007.137364

[24] Figueroa JD , Terry MB , Gammon MD , et al. Cigarette smoking, body mass index, gastro‐esophageal reflux disease, and non‐steroidal anti‐inflammatory drug use and risk of subtypes of esophageal and gastric cancers by P53 overexpression. Cancer Causes Control. 2009;20:361‐368. 18989634 10.1007/s10552-008-9250-6 PMC2726999

[25] Islami F , Sheikhattari P , Ren J , Kamangar F . Gastric atrophy and risk of oesophageal cancer and gastric cardia adenocarcinoma—a systematic review and meta‐analysis. Ann Oncol. 2010;22:754‐760. 20860989 10.1093/annonc/mdq411

[26] Mukaisho K‐I , Nakayama T , Hagiwara T , Hattori T , Sugihara H . Two distinct etiologies of gastric cardia adenocarcinoma: interactions among pH, Helicobacter pylori, and bile acids. Front Microbiol. 2015;6:412. 26029176 10.3389/fmicb.2015.00412 PMC4426758

[27] Falk GW . Updated guidelines for diagnosing and managing barrett esophagus. Gastroenterol Hepatol (NY). 2016;12:449‐451. PMC4969783 27489529

[28] Lee YY , McColl KE . Pathophysiology of gastroesophageal reflux disease. Best Pract Res Clin Gastroenterol. 2013;27:339‐351. 23998973 10.1016/j.bpg.2013.06.002

[29] Wu AH , Tseng CC , Bernstein L . Hiatal hernia, reflux symptoms, body size, and risk of esophageal and gastric adenocarcinoma. Cancer. 2003;98:940‐948. 12942560 10.1002/cncr.11568

[30] Lee YY , Wirz AA , Whiting JG , et al. Waist belt and central obesity cause partial hiatus hernia and short‐segment acid reflux in asymptomatic volunteers. Gut. 2014;63:1053‐1060. 24064007 10.1136/gutjnl-2013-305803

[31] Robertson EV , Derakhshan MH , Wirz AA , et al. Central obesity in asymptomatic volunteers is associated with increased intrasphincteric acid reflux and lengthening of the cardiac mucosa. Gastroenterology. 2013;145:730‐739. 23796455 10.1053/j.gastro.2013.06.038

[32] Watari J , Chen N , Amenta PS , et al. Helicobacter pylori associated chronic gastritis, clinical syndromes, precancerous lesions, and pathogenesis of gastric cancer development. World J Gastroenterol. 2014;20:5461‐5473. 24833876 10.3748/wjg.v20.i18.5461 PMC4017061

[33] Helicobacter and Cancer Collaborative Group . Gastric cancer and Helicobacter pylori: a combined analysis of 12 case control studies nested within prospective cohorts. Gut. 2001;49:347‐353. 11511555 10.1136/gut.49.3.347 PMC1728434

[34] Pourfarzi F , Whelan A , Kaldor J , Malekzadeh R . The role of diet and other environmental factors in the causation of gastric cancer in Iran–a population based study. Int J Cancer. 2009;125:1953‐1960. 19569234 10.1002/ijc.24499

[35] Cavaleiro‐Pinto M , Peleteiro B , Lunet N , Barros H . Helicobacter pylori infection and gastric cardia cancer: systematic review and meta‐analysis. Cancer Causes Control. 2011;22:375‐387. 21184266 10.1007/s10552-010-9707-2

[36] Shakeri R , Malekzadeh R , Nasrollahzadeh D , et al. Multiplex H. pylori serology and risk of gastric cardia and non‐cardia adenocarcinomas. Cancer Res. 2015;75:4876‐4883. 26383162 10.1158/0008-5472.CAN-15-0556 PMC4792189

[37] Bakhti SZ , Latifi‐Navid S , Zahri S , Bakhti FS , Hajavi N , Yazdanbod A . Are Helicobacter pylori highly cytotoxic genotypes and cardia gastric adenocarcinoma linked? Lessons from Iran. Cancer Biomark. 2017;21:235‐246. 29036792 10.3233/CBM-170701 PMC13075733

[38] Bakhti SZ , Latifi‐Navid S , Zahri S , Yazdanbod A Inverse association of Helicobacter pylori cagPAI genotypes with risk of cardia and non‐cardia gastric adenocarcinoma. Cancer Med. 2019;1‐10. 10.1002/cam4.2390 PMC6712521 31273955

[39] Cancer Genome Atlas Research Network . Comprehensive molecular characterization of gastric adenocarcinoma. Nature. 2014;513:202‐209. 25079317 10.1038/nature13480 PMC4170219

[40] Lim B , Kim JH , Kim M , Kim SY . Genomic and epigenomic heterogeneity in molecular subtypes of gastric cancer. World J Gastroenterol. 2016;22:1190‐1201. 26811657 10.3748/wjg.v22.i3.1190 PMC4716030

[41] Chia NY , Tan P . Molecular classification of gastric cancer. Ann Oncol. 2016;27:763‐769. 26861606 10.1093/annonc/mdw040

[42] Cristescu R , Lee J , Nebozhyn M , et al. Molecular analysis of gastric cancer identifies subtypes associated with distinct clinical outcomes. Nat Med. 2015;21:449‐456. 25894828 10.1038/nm.3850

[43] Dunne PD , McArt DG , Bradley CA , et al. Challenging the cancer molecular stratification dogma: intratumoral heterogeneity undermines consensus molecular subtypes and potential diagnostic value in colorectal cancer. Clin Cancer Res. 2016;22:4095‐4104. 27151745 10.1158/1078-0432.CCR-16-0032

[44] Garattini SK , Basile D , Cattaneo M , et al. Molecular classifications of gastric cancers: novel insights and possible future applications. World J Gastrointest Oncol. 2017;9:194‐208. 28567184 10.4251/wjgo.v9.i5.194 PMC5434387

[45] Uhlik MT , Liu J , Falcon BL , et al. Stromal‐based signatures for the classification of gastric cancer. Cancer Res. 2016;76:2573‐2586. 27197264 10.1158/0008-5472.CAN-16-0022

[46] Park S , Supek F , Lehner B . Systematic discovery of germline cancer predisposition genes through the identification of somatic second hits. Nat Commun. 2018;9:2601. 29973584 10.1038/s41467-018-04900-7 PMC6031629

[47] Carter H , Marty R , Hofree M , et al. Interaction landscape of inherited polymorphisms with somatic events in cancer. Cancer Discov. 2017;7:410‐423. 28188128 10.1158/2159-8290.CD-16-1045 PMC5460679

[48] Hu N , Wang Z , Song X , et al. Genome‐wide association study of gastric adenocarcinoma in Asia: a comparison of associations between cardia and non‐cardia tumours. Gut. 2016;65:1611‐1618. 10.1136/gutjnl-2015-309340 26129866 PMC5568652

[49] Nath S , Mukherjee P . MUC1: a multifaceted oncoprotein with a key role in cancer progression. Trends Mol Med. 2014;20:332‐342. 24667139 10.1016/j.molmed.2014.02.007 PMC5500204

[50] Abnet CC , Freedman ND , Hu N , et al. A shared susceptibility locus in PLCE1 at 10q23 for gastric adenocarcinoma and esophageal squamous cell carcinoma. Nat Genet. 2010;42:764‐767. 20729852 10.1038/ng.649 PMC2947317

[51] Wang LD , Zhou FY , Li XM , et al. Genome‐wide association study of esophageal squamous cell carcinoma in Chinese subjects identifies susceptibility loci at PLCE1 and C20orf54. Nat Genet. 2010;42:759‐763. 20729853 10.1038/ng.648

[52] Li D , Wu C , Cai Y , Liu B . Association of NFKB1 and NFKBIA gene polymorphisms with susceptibility of gastric cancer. Tumor Biol. 2017;39:1010428317717107 10.1177/1010428317717107 28670959

[53] Persson C , Canedo P , Machado JC , El‐Omar EM , Forman D . Polymorphisms in inflammatory response genes and their association with gastric cancer: a HuGE systematic review and meta‐analyses. Am J Epidemiol. 2011;173:259‐270. 21178102 10.1093/aje/kwq370 PMC3105271

[54] Guo W , Cui Y , Fang S , Li Y , Wang N , Zhang J . Association of polymorphisms of p21cip1 and p27kip1 genes with susceptibilities of esophageal squamous cell carcinoma and gastric cardiac adenocarcinoma. Ai zheng. 2006;25:194‐199. 16480585

[55] He L , Shen Y . MTHFR C677T polymorphism and breast, ovarian cancer risk: a meta‐analysis of 19,260 patients and 26,364 controls. Onco Targets Ther. 2017;10:227‐238. 28123304 10.2147/OTT.S121472 PMC5229257

[56] Miao X , Xing D , Tan W , Qi J , Lu W , Lin D . Susceptibility to gastric cardia adenocarcinoma and genetic polymorphisms in methylenetetrahydrofolate reductase in an at‐risk Chinese population. Cancer Epidemiol Prev Biomark. 2002;11:1454‐1458. 12433726

[57] Miao X , Zhang X , Zhang L , et al. Adenosine diphosphate ribosyl transferase and X‐ray repair cross‐complementing 1 polymorphisms in gastric cardia cancer. Gastroenterology. 2006;131:420‐427. 16890595 10.1053/j.gastro.2006.05.050

[58] Hein DW , Doll MA , Gray K , Rustan TD , Ferguson RJ . Metabolic activation of N‐hydroxy‐2‐aminofluorene and N‐hydroxy‐2‐acetylaminofluorene by monomorphic N‐acetyltransferase (NAT1) and polymorphic N‐acetyltransferase (NAT2) in colon cytosols of Syrian hamsters congenic at the NAT2 locus. Can Res. 1993;53:509‐514. 8425184

[59] Zhang X‐M , Zhong R , Liu L , et al. Smoking and COX‐2 functional polymorphisms interact to increase the risk of gastric cardia adenocarcinoma in Chinese population. PLoS ONE. 2011;6:e21894. 21779349 10.1371/journal.pone.0021894 PMC3136492

[60] Chen W , Wu Q , Ren H . Meta‐analysis of associations between MDM2 SNP309 polymorphism and gastric cancer risk. Biomed Rep. 2014;2:105‐111. 24649079 10.3892/br.2013.181 PMC3917030

[61] Yang M , Guo Y , Zhang X , et al. Interaction of P53 Arg72Pro and MDM2 T309G polymorphisms and their associations with risk of gastric cardia cancer. Carcinogenesis. 2007;28:1996‐2001. 17638920 10.1093/carcin/bgm168

[62] Palafox M , Ferrer I , Pellegrini P , et al. RANK induces epithelial–mesenchymal transition and stemness in human mammary epithelial cells and promotes tumorigenesis and metastasis. Can Res. 2012;72:2879‐2888. 10.1158/0008-5472.CAN-12-0044 22496457

[63] Zhang W‐B , Gu H‐Y , Shi Y‐J , et al. RANK rs1805034 T>C polymorphism is associated with susceptibility to gastric cardia adenocarcinoma in a Chinese population. Oncol Res Treat. 2015;38:503‐510. 26451891 10.1159/000440855

[64] Blank C , Gajewski TF , Mackensen A . Interaction of PD‐L1 on tumor cells with PD‐1 on tumor‐specific T cells as a mechanism of immune evasion: implications for tumor immunotherapy. Cancer Immunol Immunother. 2005;54:307‐314. 15599732 10.1007/s00262-004-0593-x PMC11032914

[65] Tang W , Chen Y , Chen S , Sun B , Gu H , Kang M . Programmed death‐1 (PD‐1) polymorphism is associated with gastric cardia adenocarcinoma. Int J Clin Exp Med. 2015;8:8086. 26221374 PMC4509319

[66] Derks S , Liao X , Chiaravalli AM , et al. Abundant PD‐L1 expression in Epstein‐Barr Virus‐infected gastric cancers. Oncotarget. 2016;7:32925‐32932. 27147580 10.18632/oncotarget.9076 PMC5078063

[67] Stevens SJ , Van Ravenswaaij‐Arts C , Janssen JW , et al. MYT1L is a candidate gene for intellectual disability in patients with 2p25. 3 (2pter) deletions. Am J Med Genet A. 2011;155:2739‐2745. 10.1002/ajmg.a.34274 21990140

[68] Zhang Y , Zhu H , Zhang X , et al. Clinical significance of MYT1L gene polymorphisms in Chinese patients with gastric cancer. PLoS ONE. 2013;8:e71979. 24015200 10.1371/journal.pone.0071979 PMC3756043

[69] Zhou R‐M , Niu C‐X , Wang N , et al. XPG gene polymorphisms and the risk of gastric cardia adenocarcinoma. Genet Test Mol Biomark. 2016;20:432‐437. 10.1089/gtmb.2015.0333 27228234

[70] Jakubowska K , Pryczynicz A , Januszewska J , et al. Expressions of matrix metalloproteinases 2, 7, and 9 in carcinogenesis of pancreatic ductal adenocarcinoma. Dis Markers. 2016;2016:9895721. 27429508 10.1155/2016/9895721 PMC4939209

[71] Miao X , Yu C , Tan W , et al. A functional polymorphism in the matrix metalloproteinase‐2 gene promoter (‐1306C/T) is associated with risk of development but not metastasis of gastric cardia adenocarcinoma. Cancer Res. 2003;63:3987‐3990. 12873995

[72] Liu L , Wu C , Wang Y , et al. Association of candidate genetic variations with gastric cardia adenocarcinoma in Chinese population: a multiple interaction analysis. Carcinogenesis. 2010;32:336‐342. 21148629 10.1093/carcin/bgq264

[73] Jones PA , Laird PW . Cancer‐epigenetics comes of age. Nat Genet. 1999;21:163. 9988266 10.1038/5947

[74] Zhou SL , Cui J , Fan ZM , et al. Polymorphism of A133S and promoter hypermethylation in Ras association domain family 1A gene (RASSF1A) is associated with risk of esophageal and gastric cardia cancers in Chinese population from high incidence area in northern China. BMC Cancer. 2013;13:259. 23705663 10.1186/1471-2407-13-259 PMC3668992

[75] Guo W , Dong Z , Guo Y , Chen Z , Kuang G , Yang Z . Aberrant methylation of the CpG island of HLTF gene in gastric cardia adenocarcinoma and dysplasia. Clin Biochem. 2011;44:784‐788. 21531217 10.1016/j.clinbiochem.2011.04.006

[76] Guo W , Dong Z , He M , et al. Aberrant methylation of thrombospondin‐1 and its association with reduced expression in gastric cardia adenocarcinoma. J Biomed Biotechnol. 2010;2010:721485. 20300551 10.1155/2010/721485 PMC2838370

[77] Guo Y‐L , Zhu T‐N , Guo W , et al. Aberrant CpG island shore region methylation of CAV1 is associated with tumor progression and poor prognosis in gastric cardia adenocarcinoma. Arch Med Res. 2016b;47:460‐470. 27986126 10.1016/j.arcmed.2016.10.005

[78] Sarbia M , Geddert H , Klump B , Kiel S , Iskender E , Gabbert HE . Hypermethylation of tumor suppressor genes (p16INK4A, p14ARF and APC) in adenocarcinomas of the upper gastrointestinal tract. Int J Cancer. 2004;111:224‐228. 15197775 10.1002/ijc.20212

[79] Guo W , Dong Z , Liu S , et al. Promoter hypermethylation‐mediated downregulation of miR‐770 and its host gene MEG3, a long non‐coding RNA, in the development of gastric cardia adenocarcinoma. Mol Carcinog. 2017;56:1924‐1934. 28345805 10.1002/mc.22650

[80] Guo W , Lv P , Liu S , et al. Aberrant methylation‐mediated downregulation of long noncoding RNA C5orf66‐AS1 promotes the development of gastric cardia adenocarcinoma. Mol Carcinog. 2018;57(7):854‐865. 10.1002/mc.22806 29566283

[81] Guo Y , Guo W , Chen Z , Kuang G , Yang Z , Dong Z . Hypermethylation and aberrant expression of Wnt‐antagonist family genes in gastric cardia adenocarcinoma. Neoplasma. 2011;58:110. 21275459 10.4149/neo_2011_02_110

[82] Guo W , Dong Z , Guo Y , Kuang G , Yang Z , Chen Z . Detection of promoter hypermethylation of the CpG island of E‐cadherin in gastric cardiac adenocarcinoma. Eur J Med Res. 2009;14:453‐458. 19748854 10.1186/2047-783X-14-10-453 PMC3352230

[83] Wang X , Kang GH , Campan M , et al. Epigenetic subgroups of esophageal and gastric adenocarcinoma with differential GATA5 DNA methylation associated with clinical and lifestyle factors. PLoS ONE. 2011;6:e25985. 22028801 10.1371/journal.pone.0025985 PMC3197593

[84] Hanai J‐I , Cao P , Tanksale P , et al. The muscle‐specific ubiquitin ligase atrogin‐1/MAFbx mediates statin‐induced muscle toxicity. J Clin Investig. 2007;117:3940‐3951. 17992259 10.1172/JCI32741 PMC2066198

[85] Guo W , Zhang M , Guo Y , Shen S , Guo X , Dong Z . FBXO32, a new TGF‐β/Smad signaling pathway target gene, is epigenetically inactivated in gastric cardia adenocarcinoma. Neoplasma. 2015;62:646‐657. 25997968 10.4149/neo_2015_078

[86] Guo W , Dong Z , Guo Y , et al. Aberrant methylation and loss expression of RKIP is associated with tumor progression and poor prognosis in gastric cardia adenocarcinoma. Clin Exp Metas. 2013;30:265‐275. 10.1007/s10585-012-9533-x 22983529

[87] Espinosa‐Parrilla Y , Muñoz X , Bonet C , et al. Genetic association of gastric cancer with miRNA clusters including the cancer‐related genes MIR29, MIR25, MIR93 and MIR106: results from the EPIC‐EURGAST study. Int J Cancer. 2014;135:2065‐2076. 24643999 10.1002/ijc.28850

[88] Gu J , Li Y , Fan L , et al. Identification of aberrantly expressed long non‐coding RNAs in stomach adenocarcinoma. Oncotarget. 2017;8:49201. 28484081 10.18632/oncotarget.17329 PMC5564761

[89] Guo W , Dong Z , Shi Y , et al. Methylation‐mediated downregulation of long noncoding RNA LOC100130476 in gastric cardia adenocarcinoma. Clin Exp Metas. 2016;33:497‐508. 10.1007/s10585-016-9794-x 27189370

[90] Wang Y , Feng X , Jia R , et al. Microarray expression profile analysis of long non‐coding RNAs of advanced stage human gastric cardia adenocarcinoma. Mol Genet Genom. 2014b;289:291‐302. 10.1007/s00438-013-0810-4 24414129

[91] Li S , Zhu J , Li J , Li S , Li B . MicroRNA‐141 inhibits proliferation of gastric cardia adenocarcinoma by targeting MACC1. Archiv Med Sci. 2018;14:588‐596. 10.5114/aoms.2017.68757 PMC5949917 29765447

[92] Liu W , Dong Z , Liang J , et al. Downregulation of potential tumor suppressor miR‐203a by promoter methylation contributes to the invasiveness of gastric cardia adenocarcinoma. Cancer Invest. 2016;34:506‐516. 27791400 10.1080/07357907.2016.1242010

[93] Wang S , Lv C , Jin H , et al. A common genetic variation in the promoter of miR‐107 is associated with gastric adenocarcinoma susceptibility and survival. Mutat Res. 2014;769:35‐41. 25771723 10.1016/j.mrfmmm.2014.07.002

[94] Gao S , Zhou F , Zhao C , et al. Gastric cardia adenocarcinoma microRNA profiling in Chinese patients. Tumor Biol. 2016;37:9411‐9422. 10.1007/s13277-016-4824-5 26781873

[95] Sun LL , Li WD , Lei FR , Li XQ . The regulatory role of microRNAs in angiogenesis‐related diseases. J Cell Mol Med. 2018;22(10):4568‐4587. 29956461 10.1111/jcmm.13700 PMC6156236

[96] Conti DV , Wang K , Sheng X , et al. Two novel susceptibility loci for prostate cancer in men of African ancestry. J Natl Cancer Inst. 2017;109:1‐5. 10.1093/jnci/djx084 PMC5448553 29117387

[97] Popejoy AB , Fullerton SM . Genomics is failing on diversity. Nature. 2016;538:161‐164. 27734877 10.1038/538161a PMC5089703

